# Pharmacogenetics of Tenofovir Renal Toxicity in HIV-positive Southern Africans

**DOI:** 10.1097/FPC.0000000000000491

**Published:** 2023-04-24

**Authors:** Somila Mateza, Yuki Bradford, Gary Maartens, Simiso Sokhela, Nomathemba C. Chandiwana, Willem D.F. Venter, Frank A. Post, Marylyn D. Ritchie, David W. Haas, Phumla Sinxadi

**Affiliations:** 1 Division of Clinical Pharmacology, Department of Medicine, University of Cape Town, Cape Town, South Africa; 2 Department of Genetics, University of Pennsylvania, Perelman School of Medicine, Philadelphia, PA, USA; 3 Wellcome Centre for Infectious Diseases Research in Africa, Institute of Infectious Disease and Molecular Medicine, University of Cape Town, Cape Town, South Africa; 4 Ezintsha, Faculty of Health Sciences, University of the Witwatersrand, Johannesburg, South Africa; 5 HIV and Infectious Diseases - King's College Hospital NHS Foundation Trust, London, UK; 6 School of Immunology and Microbial Sciences - King’s College London, London, UK; 7 Institute for Biomedical Informatics, University of Pennsylvania Perelman School of Medicine, Philadelphia, Pennsylvania, USA; 8 Department of Medicine, Vanderbilt University Medical Center, Nashville, Tennessee, USA; 9 Department of Internal Medicine, Meharry Medical College, Nashville, Tennessee, USA

**Keywords:** Tenofovir disoproxil fumarate, tenofovir alafenamide fumarate, renal toxicity, HIV, pharmacogenetics

## Abstract

**Objective:**

Renal toxicity is more likely with tenofovir disoproxil fumarate (TDF) than with tenofovir alafenamide (TAF). We investigated whether polymorphisms in genes relevant to tenofovir, TDF, and TAF disposition affect renal toxicity risk among HIV-positive Southern Africans.

**Methods:**

Adults randomized to initiate TAF or TDF in the dolutegravir arms of the ADVANCE trial were studied. Outcomes were change from week 4 to 48 in estimated glomerular filtration rate (eGFR), and from baseline to week 48 in urine retinol binding protein and urine β_2_-microglobulin, adjusted for urinary creatinine (uRBP/Cr and uB2M/Cr). Associations were assessed using multivariable linear regression models. Primary analyses prioritized 14 polymorphisms previously reported to be associated with tenofovir disposition or renal outcomes, and all polymorphisms in 14 selected genes. We also explored genome-wide associations.

**Results:**

Analyses included 336 ADVANCE participants. Among 14 polymorphisms of primary interest, the lowest P-values for change in eGFR, uRBP/Cr and uB2M/Cr were *ABCC4* rs899494 (P=0.022), *ABCC10* rs2125739 (P=0.07), and *ABCC4* rs1059751 (P=0.0088), respectively. In genes of interest, the lowest P-values were *ABCC4* rs4148481 (P=1.3x10^-4^), rs691857 (P=3.9x10^-4^), and *PKD2* rs72659631 (P=1.1x10^-3^, respectively. None withstood correction for multiple testing. Genome-wide, the lowest P-values were *COL27A1* rs1687402 (P=3.4x10^-9^), *CDH4* rs66494466 (P=5.6x10^-8^), and *ITGA4* rs3770126 (P=6.1x10^-7^), respectively.

**Conclusions:**

Two polymorphisms, *ABCC4* rs899494 and *ABCC4* rs1059751, were nominally associated with change in eGFR and uB2M/Cr, respectively, albeit in the opposite direction of previous reports. A *COL27A1* polymorphism was genome-wide significantly associated with change in eGFR. These findings enhance our understanding of the genetics of tenofovir-associated renal toxicity.

## Introduction

Tenofovir disoproxil fumarate (TDF), a prodrug of tenofovir, is recommended by the World Health Organization as part of first-line antiretroviral therapy (ART) regimens (1). In 2015, the United States Food and Drug Administration approved a second tenofovir prodrug, tenofovir alafenamide fumarate (TAF), for inclusion in ART regimens (2). Compared to TDF, TAF is as effective but has a more favourable renal and bone safety profile (3–5). The prescribing shift from TDF to TAF has been driven, in part, to avoid TDF renal tubular toxicity (6–10). The severity of renal dysfunction with TDF correlates with higher plasma tenofovir concentrations (11). Plasma tenofovir concentrations with TAF are approximately 90% lower than those with TDF (3, 5).

TDF undergoes esterase hydrolysis, which removes two ester groups to form tenofovir, while TAF undergoes hydrolysis by intracellular cathepsin A to form tenofovir (12). Tenofovir is renally eliminated by glomerular filtration and tubular secretion. Clearance in the proximal tubule is controlled by membrane transport proteins. Tenofovir is actively transported into proximal renal tubular cells by organic anion transporters 1 and 2 (OAT1 and OAT2, encoded by *SLC22A6* and *SLC22A7*, respectively), and transported out of these cells by efflux transporters including multidrug resistant protein 2 and 4 (MRP2 and MRP4, encoded by *ABCC2* and *ABCC4*, respectively) (13, 14). Genetic variants in these transporter genes have been postulated to increase risk of renal toxicity by favouring intracellular accumulation of tenofovir (11, 15, 16).

Studies of tenofovir toxicity in African populations have shown inconsistent results (17). Some found tenofovir-containing regimens to be without substantial renal toxicity (18–20), while others reported increased renal toxicity and recommend monitoring of renal function (21, 22). Candidate gene studies from various populations have sought to associate renal transporter polymorphisms with TDF renal toxicities among individuals treated for HIV. However, most did not include African populations. A study of 115 patients in Spain receiving TDF-containing regimens found an association between renal tubular dysfunction and homozygosity for *ABCC2* –24C/C (rs717620) (23). Similarly, an association between TDF-induced renal tubular dysfunction and two *ABCC2* polymorphisms (rs717620 and rs2273697) was reported in a Japanese population (24). Among 501 participants randomized to TDF-containing regimens in AIDS Clinical Trials Group protocol A5202 (25), a *SLC22A2* polymorphism (rs3127573) was associated with more favourable creatinine clearance among African-American participants (26). A study of Ghanaians receiving TDF-containing regimens suggested an associated between an *ABCC10* polymorphism (rs2125739) and worsening renal function (27).

Because of inconsistent data from pharmacogenomic studies and the limited data regarding the pharmacogenetics of TDF toxicity in Africans, who have the highest genetic diversity worldwide, we evaluated whether laboratory markers of renal tubular injury were associated with polymorphisms in genes involved in TAF, TDF and tenofovir metabolism and transport among ART-naïve Southern Africans who had been randomized to initiate either TDF- or TAF-containing regimens in a prospective clinical trial.

## Methods

### Ethics

The present study was conducted in accordance with the Declaration of Helsinki and the ADVANCE protocol WRHI 060 (NCT03122262) received ethics and regulatory approvals from the Wits Human Research Ethics Committee (REF 160606B) and the South African Health Products Regulatory Authority (REF 20160620), respectively. Ethics approval for the pharmacogenetics sub-study was also granted by the University of Cape Town Health Sciences Human Research Ethics (REF 571/2019). Written informed consent for genetic research was obtained from study participants.

### Study population

The ADVANCE study was a 96-week, randomized, phase 3 non-inferiority clinical trial which enrolled 1053 HIV-positive participants living in Johannesburg, South Africa. The ADVANCE trial has been previously described in detail (28). In brief, participants in ADVANCE were randomly assigned 1:1:1 to receive: 1) TAF, dolutegravir, and emtricitabine; 2) TDF, dolutegravir, and emtricitabine; or 3) TDF, efavirenz, and emtricitabine (28). Eligible participants were at least 12 years of age, weighed at least 40 kg, had creatinine clearances greater than 60 mL per minute, were ART naïve within 6 months before entry, were not pregnant, did not have tuberculosis at study start (28). Among 702 participants randomised to receive either TAF, dolutegravir and emtricitabine or TDF, dolutegravir and emtricitabine, 340 (48%) consented for genetic research.

### Study design

We assessed change in estimated glomerular filtration rate (eGFR), urinary retinol binding protein/creatinine (uRBP/Cr) and urinary β_2_-microglobulin/creatinine (uB2M/Cr) ratios as complementary biomarkers of renal injury and tubular toxicity.

We *a priori* selected 23 polymorphisms previously reported to have, or investigated for, an association with tenofovir disposition and tubular dysfunction (*ABCB1* rs1045642, rs2032582, and rs1128503 (29); *ABCC2* rs717620, rs2273697, rs17222723, rs8187707, and rs8187710 (15, 29) ; *ABCC10* rs9349256 and rs2125739 (30); *ABCC4* rs1059751, rs3742106, rs1751034, rs1557070, and rs899494 (23, 29); *ABCG2* rs2231142 (31); *AK2* rs12116440, rs143825456, rs148421308, and rs6175965 (32); *SLC22A2* rs3127573 (26) ; *SLC22A6* rs11568626 (33) ; and *SLC22A11* rs11231809 (23)). We then considered all available polymorphism data between transcription start and end position (± 50 kB) in 14 candidate genes relevant to tenofovir, TAF or TDF disposition (*ABCB1*, *ABCC10*, *ABCC2*, *ABCC4*, *ABCG2*, *AK2*, *AK3*, *CES1*, *CYP3A4*, *NME1*, *SLC22A2*, *SLC22A6*, *SLC22A8* and *SLC22A11*) (26, 34).

Beyond these polymorphisms, we used a stepwise approach to prioritize sets of polymorphisms to interrogate to decrease the burden of multiple comparisons. We surmised that polymorphisms previously and strongly associated with at least one drug-related phenotype, or that have been significantly genome-wide associated with any trait, are most likely to be true associations. We explored all polymorphisms from the Pharmacogenomics Knowledgebase (PharmGKB) that were associated with any drug at levels of evidence of 1 (preponderance of evidence shows an association, replicated in multiple cohorts, and preferably with strong effect size) or 2 (moderate evidence of association, replicated but some studies may not show statistical significance, or with small effect size) (accessed 10 February 2021)(34), and all NHGRI-EBI GWAS Catalog polymorphisms with p-values less than 5.0 x10^-8^ for any trait (accessed 10 February 2021)(35). We prioritised polymorphisms common to both PharmGKB and the GWAS Catalog, considering these to have the most robust evidence for true associations. We secondarily explored all polymorphisms from PharmGKB and the GWAS Catalog (based on criteria described above), and all polymorphisms in our imputed genome-wide genotype data.

### Genetic Polymorphisms

Whole blood labelled with coded identifiers was stored and DNA extraction performed at the Sydney Brenner Institute for Molecular Bioscience at the University of the Witwatersrand, using the salting out method as described elsewhere (36). Genotyping with the Illumina Infinium Multi-Ethnic Global BeadChip (MEGA^EX^) was done at Vanderbilt Technologies for Advanced Genomics (VANTAGE) in Nashville, Tennessee, USA. Post-genotype quality control included sex checks, call rates by marker and sample, identity by descent (IDB) plots, assessment for batch effects, concordance between duplicate samples, and HapMap controls.

Quality control steps were performed using PLINK version 1.9. (37)Genotyping efficiency per participant was >95% in all samples. Markers with genotyping efficiency <95% were excluded, as were those with minor allele frequencies (MAF) <5%. We excluded 21 samples with overall genotyping call rates <95%. After quality control, data were imputed using the TOPMed reference panel after transforming to genome build 38 using liftOver and stratification by chromosome to parallelize the imputation process. (38, 39)For each chromosome in each phase, 100% concordance with genotyped data was assessed. Polymorphisms with imputation scores <0.3, genotyping call rates <99%, minor allele frequency (MAF) <0.05, or Hardy-Weinberg Equilibrium (HWE) p-values <1.0x10^-8^ were excluded. To control for population stratification, we used Eigenstrat/ Eigensoft package 6.0.1 to estimate principal components.

### Clinical and laboratory data

Clinical data from the ADVANCE trial were collected at screening, enrolment, weeks 4 and 12, and every 12 weeks until week 96 (28). Estimated glomerular filtration rate was calculated using the Chronic Kidney Disease Epidemiology Collaboration (CKD-EPI) formula (40). Change in eGFR from week 4 to week 48 (ΔeGFR_4-48_) was used in analysis. Week 4 was used as baseline because dolutegravir increases serum creatinine concentrations by inhibiting tubular secretion of creatinine, which plateaus by week 4 (41, 42). The low molecular weight proteins RBP and B2M were selected because their increases in urine are early indicators of renal tubular dysfunction or toxicity (42). These markers were calculated as ratios to urine creatinine (ΔRBP/Cr_0-48_ and ΔB2M/Cr_0-48_, respectively).

The Shapiro–Wilk test was used to assess normality, with P<0.05 considered statistically significant. Clinical data not normally distributed were described with frequencies, medians, and interquartile ranges (IQR). Natural logarithmic transformations were done on uRBP/Cr and uB2M/Cr to approximate normality.

### Association analyses

Multivariable linear regression analyses were performed to evaluate associations between genetic polymorphisms and each renal outcome. Covariates included baseline age, sex, study arm (TAF or TDF), and screening body mass index (BMI), log_10_ plasma HIV-1 RNA, CD4 T-cell count, and the first 3 principal components to account for population substructure. The inclusion of cotrimoxazole use as a covariate was also examined. Our primary objective was to identify genetic associations with renal toxicity, rather than to characterize toxicity differences between TDF and TAF on kidney biomarkers. We therefore combined both study arms in our analyses while adjusting for randomization arm. Bonferroni correction was used to account for multiple testing, with a cut-off of 0.05 divided by number of polymorphisms tested in each targeted analysis, and P = 5.0x10^−8^ for genome-wide analyses. For genome-wide significant associations we examined how well these would cross-replicate internally by randomly dividing the dataset into five groups of near equal size. We tested for association on four-fifths (training set), and for replication on the remaining one-fifth (testing set). We repeated this a total of five times, once for each training-testing set pair, and assessed how consistently the association replicated.

## Results

Of the 702 ART-naïve participants who were randomly assigned to the two dolutegravir-containing arms of ADVANCE, 336 were included in our study: 173 (51.5%) in the TAF group and 163 (48.5%) in the TDF group. All participants were Black Southern Africans (55.6% South African, 38.4%, Zimbabwean, and 6% from Lesotho, Malawi, Mozambique, Swaziland, or Zambia), median age was 32 years, most were female, and median BMI was 23.4 kg/m^2^. Baseline characteristics were similar between the TAF and TDF groups (Table 1).

Regarding changes in biomarkers, among participants in the combined TAF and TDF arms the median (IQR) ΔeGFR_4-48_ was 1.74 (-11.43 to 6.32) mL/min/1.73m^2^, with no significant difference between the arms (p=0.27). For ΔRBP/Cr_0-48_, the median (IQR) was -1.15 (-4.49 to 1.68) mg/mmol, with a significantly higher ratio in the TDF arm (p=0.01). The median (IQR) ΔB2M/Cr_0-48_ was -0.01 (-0.02 to 0.00) mg/mmol, with no significant differences between the arms (p=0.23).

Of the 23 candidate polymorphisms, 14 passed quality control (QC) in our imputed genotype data. From 14 candidate genes relevant to tenofovir, TAF and TDF disposition (*ABCB1*, *ABCC10*, *ABCC2*, *ABCC4*, *ABCG2*, *AK2*, *AK3*, *CES1*, *CYP3A4*, *NME1*, *SLC22A2*, *SLC22A6*, *SLC22A8* and *SLC22A11* (± 50 kB)), we identified 6304 polymorphisms. From PharmGKB, 173 polymorphisms were previously associated with at least one medication-related phenotype, and 31 passed QC in our imputed genotype data. From NHGRI-EBI GWAS Catalog, of 89,716 polymorphisms previously associated with any trait at P < 5.0 x10^-8^ in at least one published study, 59,575 passed QC our imputed genotyped data. The remaining polymorphisms did not meet MAF, imputation score, or Hardy-Weinberg Equilibrium (HWE) cut-offs.

### Genetic associations with change in estimated glomerular filtration rate

**Figure 1:**
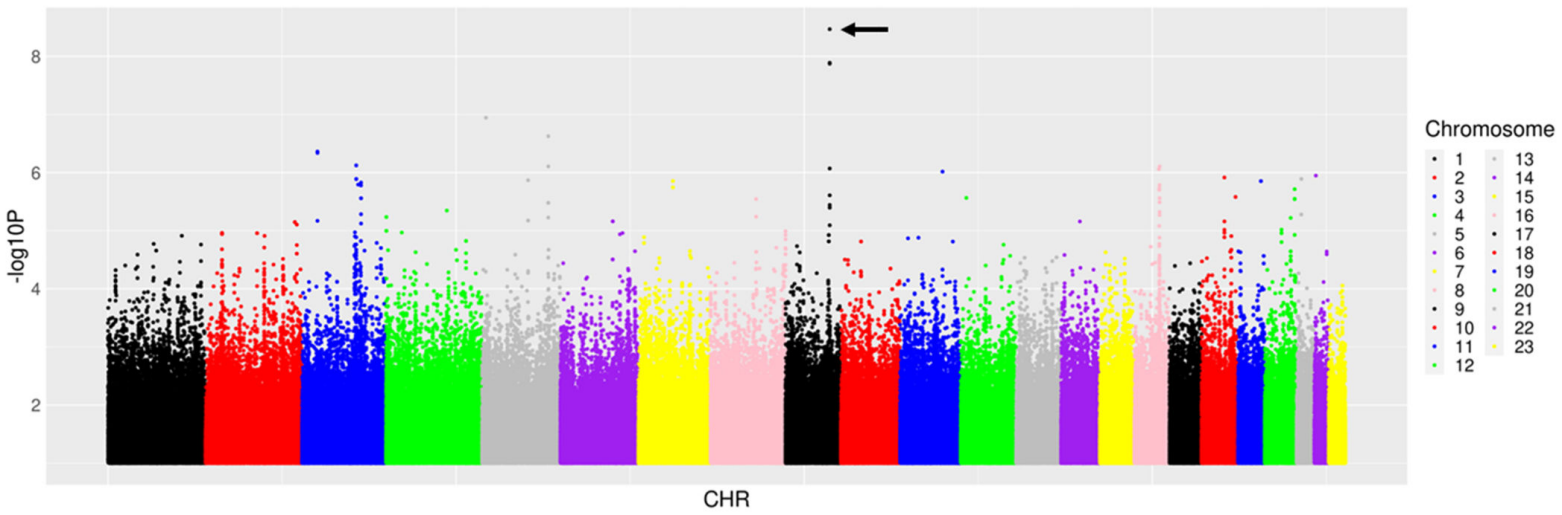
Manhattan plots of genome-wide associations with change in estimated glomerular filtration rate from week 4 to week 48. The figure shows -log_10_*P* values for association among 300 individuals who were evaluable for genetic associations. The black arrow indicates the lowest *P* value. The lowest *P* value was *COL27A1* rs168402 (*P* =3.4 × 10^−9^). Two other *COL27A1* polymorphisms in strong linkage disequilibrium with rs168402 were also genome-wide significant.

We first characterised associations with ΔeGFR_4-48_. Among 14 candidate polymorphisms previously reported to be associated with TAF, TDF and tenofovir disposition or renal toxicity, the lowest P-value for association with ΔeGFR_4-48_ was *ABCC4* rs899494 (β = 3.49, P = 0.02). Among 6304 polymorphisms in the candidate genes *ABCB1*, *ABCC10*, *ABCC2*, *ABCC4*, *ABCG2*, *AK2*, *AK3*, *CES1*, *CYP3A4*, *NME1*, *SLC22A2*, *SLC22A6*, *SLC22A8* and *SLC22A11* (± 50 kB), the lowest P-value for association with ΔeGFR_4-48_ was *ABCC4* rs4148481 (β = 5.21, P = 1.3 x10^-4^). Among 51 PharmGKB polymorphisms (of which 20 were also in the GWAS Catalog), the lowest P-value for association with ΔeGFR_4-48_ was *CYP3A5* rs776746 (β = -3.92, P = 0.031). Among 59,575 polymorphisms previously associated with any GWAS Catalog trait, the lowest P-value for association with ΔeGFR_4-48_ was *NOL4L* rs1555133 (β = 8.65, P = 1.1 x10^-5^). Considering genome-wide associations regardless of the GWAS Catalog, the lowest P-value for association with ΔeGFR_4-48_ was *COL27A1* rs1687402 (β = -9.4, P = 3.4 x 10^-9^) (Figure 1). In the above analyses for ΔeGFR_4-48_, only *COL27A1* rs1687402 withstood correction for multiple testing. Testing of rs1687402 for internal cross-replication (described in Methods) showed that, in three of five training sets (mean n = 240), this association remained genome-wide significant, and was P = 5.3 x 10^-5^ and P = 1.2 x 10^-7^ in the other two training sets. In four of five testing sets (mean n = 60), this association was nominally significant at P< 0.05, and was 0.083 in the remaining testing set. The direction of association was consistent in every set. The five lowest P-values for association with ΔeGFR4-48 from each prioritized analysis are presented in Table 2.

As a sensitivity analysis, we repeated the above analyses but based on change in eGFR from screening (rather than week 4) to week 48. In this analysis, no associations withstood correction for multiple testing. (Supplementary data available on request.) Inclusion of cotrimoxazole use as a covariate did not substantially alter genetic associations.

### Genetic associations with change in log-transformed urinary retinol binding protein/creatinine ratio

**Figure 2:**
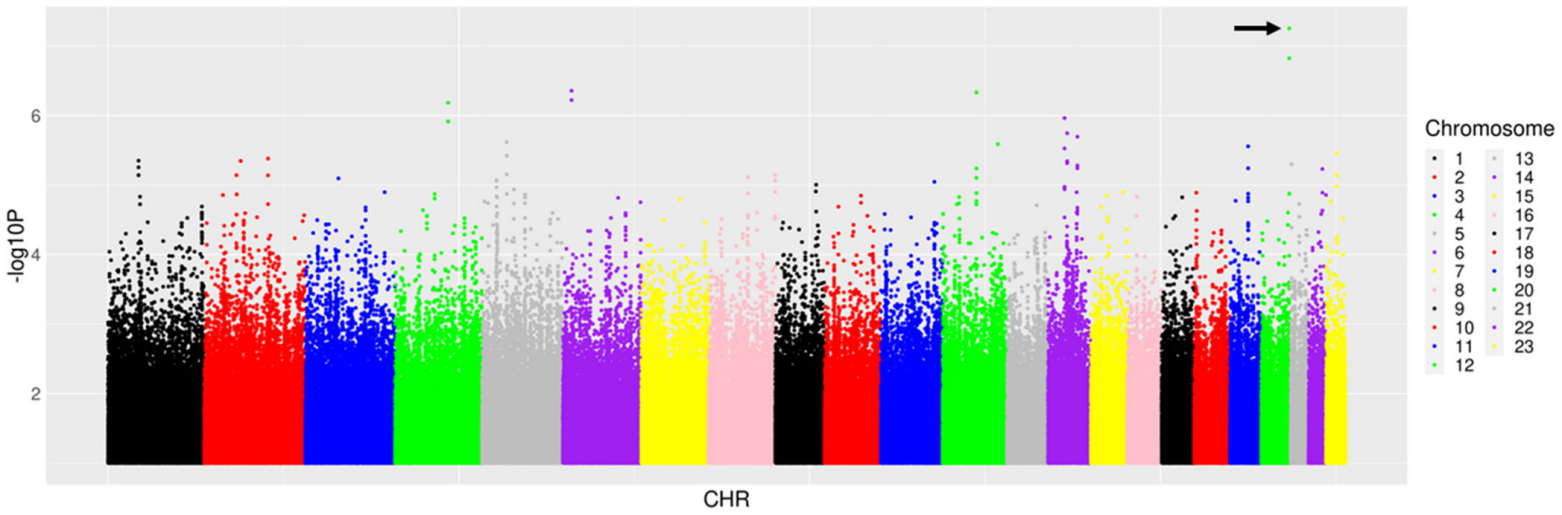
Manhattan plots of genome-wide associations with change in log-transformed urinary retinol binding protein/creatinine ratio from baseline to week 48. The figure shows -log_10_*P* values for association among 262 individuals who were evaluable for genetic associations. The black arrow indicates the lowest *P* value. The lowest *P* value was *CDH4* rs66494466 (*P* = 5.6 × 10^−8^). No polymorphisms withstood correction for multiple testing.

We then characterised associations with ΔRBP/Cr_0-48_. Among 14 candidate polymorphisms previously reported to be associated with TAF, TDF and tenofovir disposition or renal toxicity, the lowest P-value for association with ΔRBP/Cr_0-48_ was *ABCC10* rs2125739 (β = 0.19, P = 0.07). Among 6304 polymorphisms in the 14 candidate genes, the lowest P-value for association with ΔRBP/Cr_0-48_ was intergenic polymorphism rs691857 on chromosome 9 (β = -0.35, P = 3.9x10^-4^). Among 51 PharmGKB polymorphisms, the lowest P-value for association with ΔRBP/Cr_0-48_ was *APOE* rs7412 (β = -0.24, P = 0.072). Among 59,575 polymorphisms previously associated with any GWAS Catalog trait, the lowest P-value for association with ΔRBP/Cr_0-48_ was intergenic polymorphism rs3848600 on chromosome 19 (β = - 0.82, P = 1.5 x10^-5^). Considering genome-wide associations regardless of the GWAS Catalog, the lowest P-value for association with ΔRBP/Cr_0-48_ was *CDH4* rs66494466 (β = -0.70, P = 5.6 x10^-8^) (Figure 2). In the above analyses for ΔRBP/Cr_0-48_, no polymorphism withstood correction for multiple testing. The five lowest P-values for association with ΔRBP/Cr0-48 from each prioritized analysis are presented in Table 3. Inclusion of cotrimoxazole use as a covariate did not substantially alter genetic associations.

### Genetic associations with change in log-transformed urinary β_2_-microglobulin/creatinine ratio

**Figure 3:**
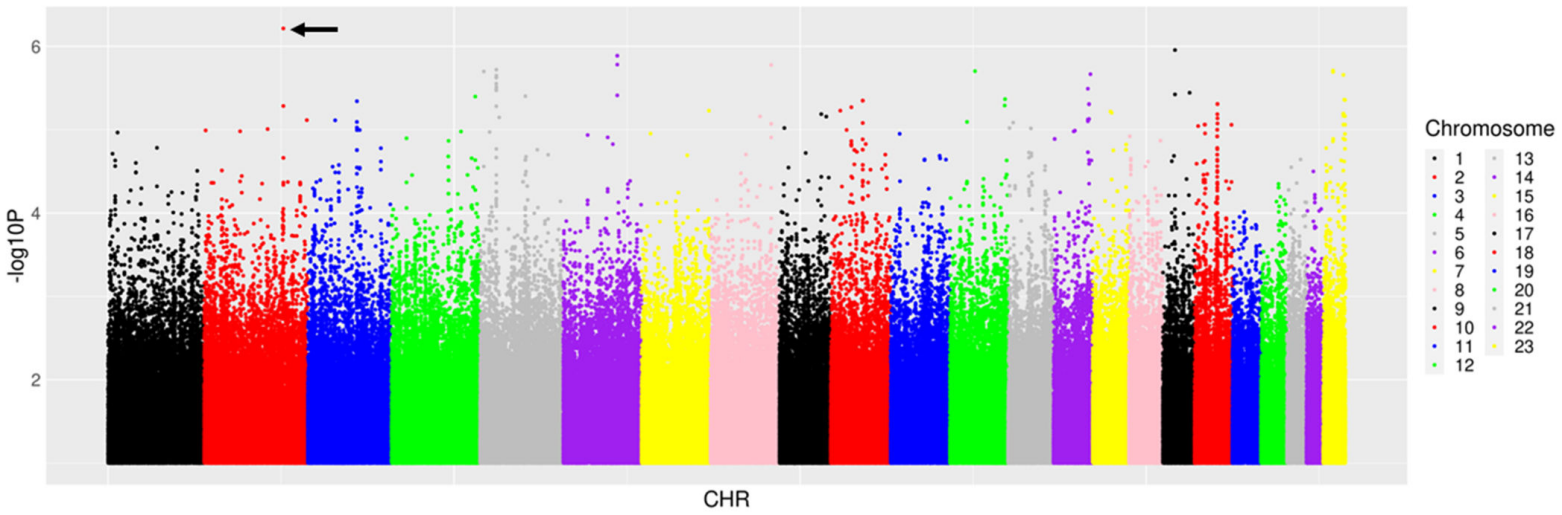
Manhattan plots of genome-wide associations with change in log-transformed urinary β_2_-microglobulin/creatinine ratio from baseline to week 48. The figure shows -log_10_*P* values for association among 290 individuals who were evaluable for genetic associations. The black arrow indicates the lowest *P* value. The lowest *P* value was *ITGA4* rs3770126 (*P* = 6.1 × 10^−7^). No polymorphisms withstood correction for multiple testing.

We next characterised associations with ΔB2M/Cr0-48. Among 14 candidate polymorphisms previously reported to be associated with TAF, TDF and tenofovir disposition or renal toxicity, the lowest P-value for association with ΔB2M:Cr_0-48_ was *ABCC4* rs1059751 (β = -0.37, P = 0.009). Among 6304 polymorphisms in the 14 candidate genes, the lowest P-value for association with ΔB2M/Cr0-48 was *PKD2* rs72659631 (β = -0.55, P = 0.001). Among 51 PharmGKB polymorphisms, the lowest P-value for association with ΔB2M/Cr_0-48_ was intergenic polymorphism rs489693 on chromosome 18 (β = -0.21, P = 0.07). Among 59,575 polymorphisms previously associated with any GWAS Catalog trait, the lowest P-value for association with ΔB2M/Cr_0-48_ was *ITG4* rs1143676 (β = -0.52, P = 2.2 x10^-5^). Considering genome-wide associations regardless of the GWAS Catalog, the lowest P-value for association with ΔB2M/Cr_0-48_ was *ITG4* rs3770126 (β = -0.79, P = 6.1 x10^-7^) (Figure 3). In the above analyses for ΔB2M/Cr0-48, no polymorphism withstood correction for multiple testing. The five lowest P-values for association with ΔB2M/Cr_0-48_ from each prioritized analysis are presented in Table 4. Inclusion of cotrimoxazole use as a covariate did not substantially alter genetic associations.

### Associations by randomized study arm

Our study included randomised arm in ADVANCE (TAF or TDF) as a covariate. In our multivariable analyses, randomized study arm was not independently associated with any of the renal outcomes examined.

## Discussion

We characterised genetic associations with renal tubular dysfunction among ART-naïve Southern African participants who were randomized to the two dolutegravir arms (plus either TAF and emtricitabine, or TDF and emtricitabine) of the ADVANCE clinical trial. Among 14 polymorphisms that we prioritized based on reported associations with TDF renal toxicity or plasma tenofovir exposure, we found nominal associations with two polymorphisms previously reported - *ABCC4* rs899494 with ΔeGFR_4-48_, and *ABCC4* rs1059751 with ΔB2M/Cr_0-48_. However, these associations did not withstand correction for multiple comparisons and were in the opposite direction to those previously reported, as discussed below. In addition, three polymorphisms in *COL27A1*, which encodes collagen type XXVII alpha 1 chain, were genome-wide associated with change in eGFR.

Our primary analyses focused on polymorphisms previously reported to be associated with TDF-related renal toxicity in other populations. Prior studies have implicated multidrug resistance proteins, encoded by *ABCC* family members, in tenofovir clearance and TDF-related renal toxicity (14, 23, 30, 43). We found that *ABCC4* rs1059751 was nominally associated with ΔB2M/Cr_0-48_ (P = 0.009). A previous report associated rs1059751 T→C (at position 4976 of *ABCC4*) with β_2_-microglobulinuria in Thai patients treated with TDF-containing ART (44). In that study, the rs1059751 C allele was associated with β_2_-microglobulinuria, with allele frequency of 0.60 in cases and 0.48 in controls. In contrast, the present study associated the rs1059751 G allele (equivalent to C allele in the Thai study) with a decrease in urinary β_2_-microglobulin (β = -0.37), with a MAF of 0.19. The negative association indicates that participants with the minor G allele in our study were less likely to experience renal tubular dysfunction (increased β_2_-microglobulinuria). We also found that *ABCC4* rs899494 G→A was nominally associated ΔeGFR_4-48_ (P = 0.02). This polymorphism was previously associated with renal phosphorus wasting in Spanish patients treated with TDF-containing ART. (23) In that study, the CC genotype was associated with worsening of the renal marker. However, for the present study, the A allele (equivalent to T allele in the Thai study) was associated with greater eGFR decline, which may be indicative of renal tubular dysfunction (45). We did not replicate associations with *ABCC2* rs2273697 and *ABCC10* rs2125739, which were previously reported to be associated with altered amino acid excretion (23) and with kidney tubular dysfunction, respectively (30).

In the present study, several *COL27A1* polymorphisms were genome-wide associated with change in ΔeGFR4-48. To help assess whether this was by chance alone, we performed internal cross-replication, which showed that the association replicated in most, but not all, paired training-testing datasets. This gene encodes collagen type XXVII alpha 1 chain (46), and mutations in *COL27A1* have been associated with Steel syndrome, knee osteoarthritis and Achilles tendinopathy (47–49). Although *COL27A1* is expressed in renal tubules (50), it is unclear how this gene might contribute mechanistically to tenofovir renal toxicity.

To identify novel polymorphisms associated with the renal outcomes, we leveraged imputed genome-wide data against the considerable knowledge generated by prior genetic association studies represented in PharmGKB and the GWAS Catalog. We reasoned that those polymorphisms associated with at least one drug-related phenotype in PharmGKB with levels of evidence of 1 or 2 (as described in Methods) or with any trait in the GWAS Catalog at P < 5.0 x10^-8^ in at least one published study, would most likely be true positives in the present study. We did not identify any such associations that withstood correction for multiple testing.

Our study has some limitations. A larger sample size would have increased power to detect associations with modest effect size, or associations with infrequent variants. We did not adjust for concomitant drugs that are potentially nephrotoxic, which may have obscured genetic associations. The study was performed exclusively in Black people living with HIV, who were previously shown to be at lower risk of renal tubular dysfunction (51) and TDF-associated renal tubulopathy (45); our results can therefore not be extrapolated to other populations. The relatively short exposure (48 weeks) and use of an unboosted third agent in this young population may have contributed to the minimal effects of tenofovir on renal tubular function, and thus our ability to identify genetic associations with the three measures of renal tubular dysfunction. In our relatively young cohort, we were unable to adjust for pre-existing hypertension, diabetes or cardiovascular disease.

In conclusion, we found associations with two polymorphisms previously reported to be associated with TDF-induced renal toxicity, although in our study the associations did not withstand correction for multiple comparisons and were in the opposite directions than previously reported. We also identified genome-wide significant associations with change in eGFR and polymorphisms in *COL27A1*. These findings contribute to our understanding of tenofovir-related renal toxicity.

## Figures and Tables

**Table 1:** Baseline characteristics of study participants by randomization arm (TAF, dolutegravir, and emtricitabine or TDF, dolutegravir, and emtricitabine)

Characteristics median [IQR ^b^]	TAF/DTG/FTC (n = 173)	THF/DTG/FTC (n = 163)	P-value ^a^
**Age (years)**	32 [27 to 38]	32 [27 to 37]	0.90
**Sex, n (%)**			0.88
**Female**	109 (63%)	104 (64%)	
**Male**	64 (37%)	59 (36%)	
**Screening BMI (kg/m^2^)**	23.6 [20.6 to 26.5]	23.1 [20.1 to 27.1]	0.98
**Screening CD4 count (cells/μl)**	334 [170 to 502]	276 [159 to 424]	0.22
**Screening HIV-1 RNA (log_10_ copies/mL)**	4.5 [3.8 to 4.9]	4.4 [3.8 to 4.9]	0.79
**Screening hepatitis B positive, n (%)**	11 (6.4%)	6 (3.7%)	0.26
**Cotrimoxazole use, n (%)**	24 (13.9%)	32 (19.6%)	0.16
**Renal biomarkers at baseline**			
**eGFR week 0 (mL/min/1.73m^2^)**	135.8 [125.4 to 142.2]	135.8 [124.4 to 142.7]	0.74
**eGFR week 4 (mL/min/1.73m^2^)**	123 [104.7 to 134.2]	117 [100.5 to 131.9]	0.05
**uRBP week 0 (μg/L)**	59 [29 to 128]	74 [30 to 140]	0.71
**uRBP/Cr week 0 (μg/mmol)**	5.6 [3.3 to 11.4]	6.0 [3.2 to 11.5]	0.77
**uB2M week 0 (μg/L)**	165.5 [87.5 to 295]	129 [70 to 302]	0.80
**uB2M/Cr week 0 (μg/mmol)**	0.014 [0.008 to 0.025]	0.014 [0.007 to 0.025]	0.66

a P-value for comparison between study groups was calculated using Wilcoxon rank-sum. P-value for sex, screening hepatitis B status, cotrimoxazole use was determined using Z-tests for proportions.

b abbreviations: IQR, interquartile range; TAF, tenofovir alafenamide fumarate; TDF, tenofovir disoproxil fumarate; DTG, dolutegravir; FTC, emtricitabine; eGFR, estimated glomerular filtration rate; RBP, retinol binding protein; B2M, Beta 2-Microglobulin; Cr, creatinine.

**Table 2:** Polymorphisms with lowest P-values for association with change in estimated glomerular filtration rate from week 4 to week 48

Polymorphism	Gene	Chromosome	Reference allele	Variant allele	MAF	Beta	P-value	N
Candidate SNPs^a^ (n = 14 polymorphisms)								
rs899494	*ABCC4*	13	G	A	0.26	3.47	0.022	300
rs1045642	*ABCB1*	7	G	A	0.12	-3.72	0.066	300
rs1557070	*ABCC4*	13	G	A	0.31	-2.55	0.082	300
rs1751034	*ABCC4*	13	T	C	0.36	1.79	0.19	300
rs2125739	*ABCC10*	6	T	C	0.27	1.87	0.18	300
Candidate Genes^b^ (n = 6304 polymorphisms)								
rs4148481	*ABCC4*	13	G	A	0.47	5.21	1.32E-4	300
rs17013743	*PKD2*	4	A	G	0.11	8.45	1.82E-4	300
rs55828993	*intergenic*	10	T	G	0.24	5.98	2.39E-4	300
rs2274403	*ABCC4*	13	A	G	0.47	4.99	2.48E-4	300
rs45590633	*ABCB4*	7	C	T	0.18	6.25	2.68E-4	300
PharmGKB and GWAS Catalog^c^ (n = 51 polymorphisms)								
rs776746	*CYP3A5*	7	T	C	0.19	-3.92	0.031	300
rs8050894	*VKORC1*	16	C	G	0.24	3.06	0.048	300
rs1800566	*NQO1*	16	G	A	0.18	3.33	0.066	300
rs2011425	*UGT1A8*	2	T	G	0.08	-3.20	0.183	300
rs11045879	*SLCO1B1*	12	T	C	0.06	3.48	0.207	300
GWAS Catalog^d^ (n = 59,575 polymorphisms)								
rs1555133	*NOL4L*	20	G	A	0.15	8.65	1.10E-5	300
rs11867022	*CNOT1*	16	T	C	0.43	-5.54	3.80E-5	300
rs2941741	*ESR1*	6	G	A	0.20	-6.83	4.90E-5	300
rs13143759	*LINC02502*	4	G	A	0.28	-5.74	5.20E-5	300
rs11184994	*LOC105378888*	1	C	T	0.44	-5.36	7.30E-5	300
Genome-wide analyses results^e^								
rs1687402	*COL27A1*	9	A	G	0.23	-9.44	3.42E-9	300
rs1687400	*COL27A1*	9	C	T	0.23	-9.21	1.30E-8	300
rs1626295	*COL27A1*	9	C	T	0.23	-9.21	1.34E-8	300
rs309421	*intergenic*	5	A	G	0.40	7.10	1.14.E-7	300
rs4958396	*LOC105378239*	5	G	T	0.29	7.39	2.37E-7	300

Covariates included baseline age, sex, study arm (TAF or TDF), and screening body mass index (BMI), log_10_ plasma HIV-1 RNA, CD4 T-cell count, and the first 3 principal components to account for population substructure.

a Significance threshold was 0.0036 for a subset of 14 SNPs.

b Significance threshold was 7.9x10^-6^ for a subset of 6304 SNPs.

c Significance threshold was 9.8x10^-4^ for a subset of 51 SNPs.

d Significance threshold was 8.4x10^-7^ for a subset of 59 575 SNPs.

e Significance threshold was 5x10^-8^ for the genome-wide analysis. The three polymorphisms in *COL27A1* were in strong linkage disequilibrium.

Abbreviations: SNP, single nucleotide polymorphism; MAF, minor allele frequency.

**Table 3:** Polymorphisms with lowest P-values associated with change in log-transformed urinary retinol binding protein/creatinine ratio from baseline to week 48

Polymorphism	Gene	Chromosome	Reference allele	Variant allele	MAF	Beta	P-value	N
Candidate SNPs^a^ (n = 14 polymorphisms)								
rs2125739	*ABCC10*	6	T	C	0.27	0.19	0.07	262
rs3742106	*ABCC4*	13	A	C	0.42	-0.16	0.13	262
rs1557070	*ABCC4*	13	G	A	0.31	0.13	0.23	262
rs1128503	*ABCB1*	7	G	A	0.08	0.22	0.25	262
rs11568626	*SLC22A6*	11	C	T	0.06	-0.18	0.41	262
Candidate Genes^b^ (n = 6304 polymorphisms)								
rs691857	*intergenic*	9	A	G	0.44	-0.35	3.87E-4	262
rs10737365	*intergenic*	1	A	C	0.07	-0.64	4.53E-4	262
rs410437	*intergenic*	9	A	G	0.32	0.38	4.58E-4	262
rs4149181	*SLC22A8*	11	G	A	0.45	0.32	2.09E-3	262
rs9524860	*ABCC4*	13	T	C	0.25	-0.38	2.18E-3	262
PharmGKB and GWAS Catalog^c^ (n = 51 polymorphisms)								
rs7412	*APOE*	19	C	T	0.17	-0.24	0.072	262
rs1800629	*intergenic*	6	G	A	0.15	-0.25	0.084	262
rs489693	*intergenic*	18	C	A	0.44	-0.17	0.11	262
rs11045879	*SLCO1B1*	12	T	C	0.07	-0.31	0.12	262
rs776746	*CYP3A5*	7	T	C	0.18	-0.20	0.14	262
GWAS Catalog^d^ (n = 59,575 polymorphisms)								
rs3848600	*Intergenic*	19	C	T	0.07	-0.82	1.50E-5	262
rs7247962	*Intergenic*	19	T	C	0.07	-0.87	1.70E-5	262
rs2952858	*LINC02465*	4	A	G	0.34	0.41	5.00E-5	262
rs12714199	*LOC105374845*	2	C	T	0.49	0.41	5.60E-5	262
rs2006092	*GGT1*	22	G	A	0.17	-0.52	8.00E-5	262
Genome-wide analyses results^e^								
rs66494466	*CDH4*	20	G	A	0.18	-0.70	5.56E-8	262
rs2086176685	*CDH4*	20	TTTGTCCTC	T	0.19	-0,67	1.51E-7	262
rs9472842	*PHACTR1*	6	C	T	0.10	-0.82	4.42E-7	262
rs7960855	*intergenic*	12	T	C	0.33	0.53	4.66E-7	262
rs9472865	*PHACTR1*	6	T	C	0.10	-0.80	6.01E-7	262

Covariates included baseline age, sex, study arm (TAF or TDF), and screening body mass index (BMI), log_10_ plasma HIV-1 RNA, CD4 T-cell count, and the first 3 principal components to account for population substructure.

a Significance threshold was 0.0036 for a subset of 14 SNPs.

b Significance threshold was 7.9x10^-6^ for a subset of 6304 SNPs.

c Significance threshold was 9.8x10^-4^ for a subset of 51 SNPs.

d Significance threshold was 8.4x10^-7^ for a subset of 59 575 SNPs.

e Significance threshold was 5x10^-8^ for the genome-wide analysis.

Abbreviations: SNP, single nucleotide polymorphism; MAF, minor allele frequency.

**Table 4:** Polymorphisms with lowest P-values associated with change in log-transformed urinary β_2_-microglobulin/creatinine ratio from baseline to week 48

Polymorphism	Gene	Chromosome	Reference allele	Variant allele	MAF	Beta	P-value	N
Candidate SNPs^a^ (n = 14 polymorphisms)								
rs1059751	*ABCC4*	13	A	G	0.19	-0.37	0.009	290
rs2273697	*ABCC2*	10	G	A	0.14	-0.30	0.05	290
rs2125739	*ABCC10*	6	T	C	0.27	0.16	0.18	290
rs1557070	*ABCC4*	13	G	A	0.31	0.16	0.21	290
rs1751034	*ABCC4*	13	T	C	0.36	0.13	0.25	290
Candidate Genes^b^ (n = 6304 polymorphisms)								
rs72659631	*PKD2*	4	C	T	0.12	-0.55	0.0011	290
rs1766896	*intergenic*	13	T	C	0.45	-0.37	0.0012	290
rs186032556	*intergenic*	11	C	A	0.07	-0.67	0.0015	290
rs95323851	*intergenic*	13	G	A	0.37	0.37	0.0017	290
rs9516561	*PKD2*	4	C	A	-0.40	0.23	0.0018	290
PharmGKB and GWAS Catalog^c^ (n = 51 polymorphisms)								
rs489693	*intergenic*	18	C	A	0.44	-0.21	0.070	290
rs776746	*CYP3A5*	7	T	C	0.19	-0.25	0.083	290
rs12979860	*IFNL4*	19	T	C	0.41	0.19	0.086	290
rs1801133	*MTHFR*	1	G	A	0.09	0.29	0.1307	290
rs1800566	*NQO1*	16	G	A	0.18	0.22	0.132	290
GWAS Catalog^d^(n = 59,575 polymorphisms)								
rs1143676	*ITGA4*	2	A	G	0.27	-0.52	2.20E-5	290
rs6508210	*DCC*	18	C	A	0.34	0.48	3.20E-05	290
rs7245004	*DCC*	18	A	C	0.22	0.54	4.30E-05	290
rs3770104	*ITGA4*	2	T	C	0.27	-0.49	4.30E-05	290
rs10031777	*ZAR1*	4	C	T	0.37	0.43	1.20E-4	290
Genome-wide analyses results^e^ *								
rs3770126	*ITGA4*	2	A	G	0.14	-0.79	6.10E-7	290

Covariates included baseline age, sex, study arm (TAF or TDF), and screening body mass index (BMI), log_10_ plasma HIV-1 RNA, CD4 T-cell count, and the first 3 principal components to account for population substructure.

a Significance threshold was 0.0036 for a subset of 14 SNPs.

b Significance threshold was 7.9x10^-6^ for a subset of 6304 SNPs.

c Significance threshold was 9.8x10^-4^ for a subset of 51 SNPs.

d Significance threshold was 8.4x10^-7^ for a subset of 59 575 SNPs.

e Significance threshold was 5x10^-8^ for the genome-wide analysis.

* We used a P-value cut-off of P < 1x10^-6^ for GWAS SNP filtering. Only 1SNP was associated with ΔB2M/Cr_0_._48_.

Abbreviations: SNP, single nucleotide polymorphism; MAF, minor allele frequency.
